# Esophageal Dilation: A Cross-Sectional Analysis of Patient Information

**DOI:** 10.7759/cureus.47080

**Published:** 2023-10-15

**Authors:** Anh Thu N Nguyen, April L Baum, Michael J Valentine, Caleb R McNab, Larissa Vollin, Carol E Kirila

**Affiliations:** 1 Internal Medicine, Kansas City University, Kansas City, USA; 2 Primary Care/Internal Medicine, Kansas City University, Kansas City, USA

**Keywords:** esophageal dysmotility, esophageal stricture, upper gi endoscopy, esophageal dilation, click-through rate, online information, readability, health literacy

## Abstract

Objective

Complications of esophageal strictures have decreased in recent years due to evolved endoscopic methods. This has primarily been through esophageal dilation. This study examines the level of readability of patient information on esophageal dilation across 40 websites found via internet search.

Methods

In this cross-sectional readability study, the content of the first 40 websites about “esophageal dilation” and “upper GI endoscopy” found via Google search was analyzed using WebFX (Harrisburg, PA), an established readability tool. Five readability indices, each having a unique mathematical formula, were used to analyze online material. Outputs were then scored and averaged together.

Results

The aggregate readability of online esophageal dilation was found to be 9.2, corresponding to a ninth-grade reading level. This average was found based on 38 unique, non-duplicated websites evaluated.

Conclusions

The information currently available on the internet regarding esophageal dilation is considered to be at a difficult reading level for an average patient. There remains a significant amount of development required in the domain of information accessibility to enhance the patient comprehension of invasive procedures they are poised to undergo. It is imperative to refine the articulation of complex procedures further to prepare patients for forthcoming medical procedures.

## Introduction

Esophageal strictures are abnormal narrowings of the esophageal lumen due to various causes, which lead to dysphagia. The incidence of esophageal strictures is associated with disorders such as gastroesophageal reflux disease (GERD), achalasia, and prior esophageal cancer. This complication increases with age at an estimated rate of approximately one per 10,000 people [1]. The most common cause of esophageal stricture is GERD. This accounts for an estimated 70% of cases [2]. A recent study on the safety and efficacy of esophageal dilation showed successful dilation in 102 of the 111 patients with peptic strictures with no mortalities [3]. Another meta-analysis reveals an overall per-dilation complication rate of 0.215% [4]. The estimated annual cost of treating esophageal strictures with endoscopic esophageal dilation is considered cost-effective to the patient, costing $19,822, while surgery costs $41,358 [5], a drastic difference that alleviates the burden of both the healthcare system and patients.

A range of modalities are at clinicians’ disposal for the treatment of esophageal strictures, with endoscopic esophageal dilation employing bougie or balloon dilators demonstrating noteworthy efficacy in the alleviation of dysphagia [1]. Esophageal dilation commences with the selection of a suitable dilator, such as a balloon dilator, a guided wire dilator, or bougies [6]. The selection of the appropriate dilator is based on multiple factors including the patient’s medical history.

One of the preferred methods for dysphagia amelioration is the utilization of the guided wire dilator. After the insertion of the guide wire through the endoscope, the endoscope is withdrawn, and the stricture may be dilated to the appropriate level. In most cases, this is no more than three times [7]. Despite its regarded safety, the procedure may cause complications. This includes potential esophageal perforation, which carries a mortality rate of approximately 20% [8]. Hence, it is crucial that healthcare providers impart comprehensive education before the procedure and after the procedure. Patient education is an essential tool to improve health outcomes.

In the United States, the Pew Research Center reported in 2013 that up to 80% of Americans searched for healthcare-related information online [9]. Of those persons, a significant number of patients did not get their conditions confirmed by a healthcare professional [9]. This tendency is exacerbated by the pervasive circulation of misleading information online, a phenomenon further exaggerated by the anonymity of contributors and the challenge of distinguishing credible sources [10]. Furthermore, despite the recommendations from the National Institutes of Health (NIH) advocating for healthcare materials to be articulated at a sixth-grade to seventh-grade reading level to enhance comprehension for the general population, this guideline is often unmet by the majority of online resources [11]. This discrepancy has been shown not to be achieved by the majority of online reading materials [11]. According to the Department of Education, approximately 43 million adults possess low literacy skills, with 26.5 million adults at level 1 reading level [12]. This discrepancy is significant given that the 2019 data highlighted that around 43 million adults in the United States demonstrate low literacy skills, including a substantial 26.5 million that exhibit a level 1 reading proficiency [12]. Consequently, the existing literacy gap, compounded by the insufficient simplification of healthcare materials available online, poses substantial barriers to achieving the optimal readability of patient educational materials and quality of informed consent. In light of this, the present study was conducted to investigate the readability levels of information concerning endoscopic esophageal dilation found online.

## Materials and methods

Evaluating readability and keyword rationale

Former studies of readability analysis in other fields were employed as methods for this study [13,14]. The topics of patient preparation, procedure, postoperative care, and complications with regard to esophageal dilation were established, and subsequent keywords were developed to encompass these topics. To gather the sample of websites, two keywords, “esophageal dilation” and “upper GI endoscopy,” were entered to conduct the search on Google search engine, as 88% of online searches were conducted using this search engine in the United States. The first 40 Uniform Resource Locators (URLs) in the English language were gathered in the sample.

Data and readability analysis

The readability of information on the endoscopic esophageal dilation procedure was assessed by the WebFX (Harrisburg, PA) readability tool, a highly recommended tool by the Search Engine Journal. Five readability indices were used, each with different scales to quantify a score based on emphases on different textual components [15,16]. Five readability indices were used: Automated Readability Index (ARI), Coleman-Liau Index (CLI), Simple Measure of Gobbledygook (SMOG), Gunning Fog Index (GFI), and Flesch-Kincaid Grade Level (FKGL). Outputs of this data were computed via Microsoft Excel (Microsoft® Corp., Redmond, WA). The validated indices are further detailed within the discussion. A summary of readability indices is found in Table 1.

**Table 1:** Readability indices, metrics used in calculations, and result units. SMOG: Simple Measure of Gobbledygook

Index	Metrics used in calculation	Unit of result
Flesch-Kincaid Grade Level	Words per sentence and syllables per word	Grade level
Gunning Fog Index	Words per sentence and complexity of words used	Grade level
SMOG Index	Complexity of words per sentence	Grade level
Coleman-Liau Index	Count of characters, words, and sentences	Grade level
Automated Readability Index	Count of characters, words, and sentences	Grade level

Health-specific click-through rate

The click-through rate (CTR) is the percentage of impressions that result in a click after an online search. It predicts the probability that a user will click on a specific link based on the order presented in the search engine results [17]. A high CTR typically deems a website to be highly relevant, whereas a low CTR deems it less relevant. However, the scoring is relative to the industry, keyword search, and number of advertisements that the website invested in. Regardless, the idea of CTR is that early search results generally have higher predictive CTR value, which simply indicates that those websites have higher viewership.

## Results

**Figure 1:**
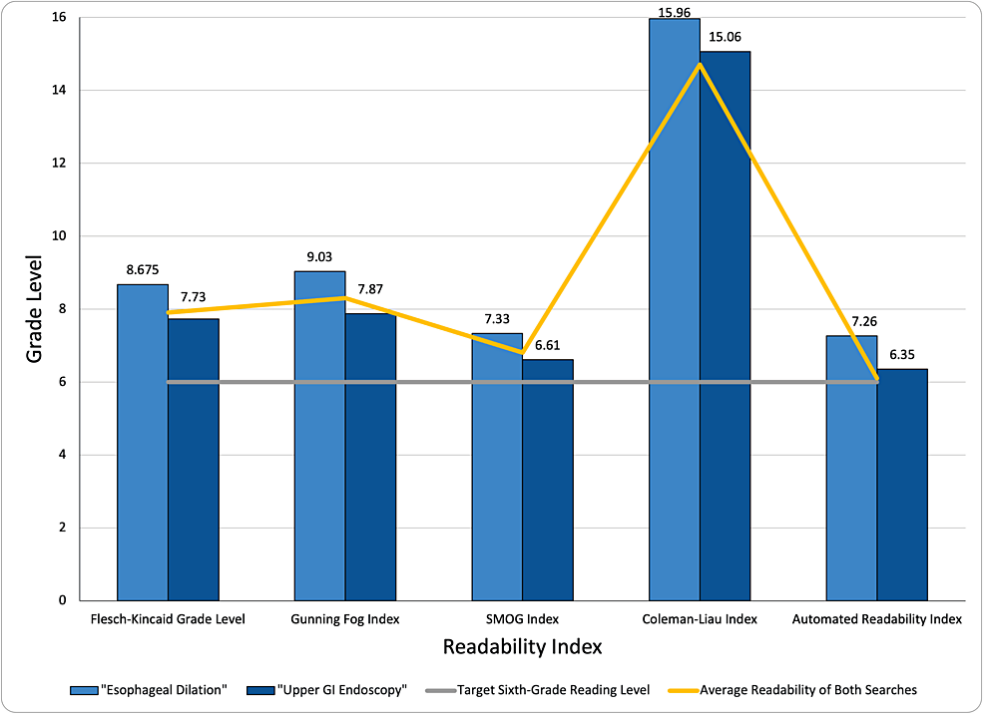
Readability of esophageal dilation information. For each readability index, results for each search criteria are displayed. Target sixth-grade reading level is represented in a linear comparison line, as well as the average readability of both searches. GI, gastrointestinal; SMOG, Simple Measure of Gobbledygook

Online material regarding esophageal dilation and upper gastrointestinal (GI) endoscopy yielded an average readability score of 9.2 (Table 2). Of the 40 websites from the initial search to be evaluated for readability regarding endoscopic esophageal dilation, 38 unique websites were included in the final data analysis, which can be seen in Table 3. The average readability grades within each indices of ARI, CLI, SMOG, GFI, and FKGL are shown in Figure 1. The computed overall readability means of “esophageal dilation” and “upper GI endoscopy” searches were 10.24 and 7.87, respectively. Both averages are above the recommended sixth-grade reading level for healthcare information per both the NIH and the American Medical Association (AMA) guidelines. Within studies pertaining to the “esophageal dilation” search, dynamed.com was the easiest to comprehend, with a readability average of 6.62, whereas the Endoscopy Center of Red Bank had the highest readability score of 12.04. Within “upper GI endoscopy” studies, Alberta had the lowest readability score of 6.72, whereas the most difficult to comprehend website was the Endoscopy Center of Red Bank. Within both studies, there were no singular websites below the recommended reading level, which can be visualized in Figure 1. Of note, the Endoscopic Center of Red Bank, being the most difficult to comprehend in both studies, was at position number 4 on the list of results upon submitting on the Google search engine. The list of websites included in the final dataset can be evaluated in Table 3.

**Table 2:** Individual search results with readability index results. The goal of a sixth-grade reading level for patient reading material is juxtaposed with each index result and average readability score. SMOG, Simple Measure of Gobbledygook; GI, gastrointestinal

Readability index	“Esophageal dilation”	“Upper GI endoscopy”	Goal	Search average
Flesch-Kincaid Grade Level	8.675	7.73	6	7.9
Gunning Fog Index	9.03	7.87	6	8.3
SMOG Index	7.33	6.61	6	6.8
Coleman-Liau Index	15.96	15.06	6	14.7
Automated Readability Index	7.26	6.35	6	6.1
Average Readability	9.651	8.724		9.1875

**Table 3:** Websites used to analyze data, numbered 1-20 for each keyword search group. Bolded websites were omitted from the data analysis due to a lack of general public accessibility. GI: gastrointestinal

Position in query	Google search number 1: “esophageal dilation”	Google search number 2: “upper GI endoscopy”
1	https://www.asge.org/home/for-patients/patient-information/understanding-eso-dilation-updated	https://www.hopkinsmedicine.org/health/treatment-tests-and-therapies/upper-gi-endoscopy
2	https://www.saintlukeskc.org/health-library/esophageal-dilation	https://www.niddk.nih.gov/health-information/diagnostic-tests/upper-gi-endoscopy
3	https://www.verywellhealth.com/esophageal-dilation-1191856	https://www.mayoclinic.org/tests-procedures/endoscopy/about/pac-20395197
4	https://endoscopycenterofredbank.com/procedure/esophageal-dilation	https://my.clevelandclinic.org/health/treatments/4957-upper-endoscopy-procedure
5	https://myhealth.alberta.ca/Health/aftercareinformation/pages/conditions.aspx?hwid=bo1337	https://patient.gastro.org/upper-gi-endoscopy/
6	https://www.chop.edu/treatments/esophageal-dilatation	https://www.uptodate.com/contents/upper-endoscopy-beyond-the-basics/print
7	https://columbiasurgery.org/conditions-and-treatments/esophageal-dilation	https://iffgd.org/manage-your-health/gi-motility-tests/upper-gi-endoscopy/
8	https://www.gpddc.com/2017/08/31/benefits-esophageal-dilation/	https://www.asahq.org/madeforthismoment/preparing-for-surgery/procedures/upper-endoscopy/
9	https://www.rwjbh.org/treatment-care/digestive-health/treatments/esophageal-dilation/	https://www.urmc.rochester.edu/encyclopedia/content.aspx?contenttypeid=92&contentid=P07717
10	https://www.insitedigestive.com/services-procedures/esophageal-dilation/	https://muschealth.org/medical-services/ddc/patients/gi-procedures/upper-endoscopy
11	https://www.sciencedirect.com/science/article/abs/pii/S0016510706003993	https://www.cancer.net/navigating-cancer-care/diagnosing-cancer/tests-and-procedures/upper-endoscopy
12	https://www.wakehealth.edu/treatment/e/esophageal-dilation	https://www.pennmedicine.org/for-patients-and-visitors/find-a-program-or-service/gastroenterology/diagnostic-testing-and-procedures/endoscopy-procedures/upper-gi-endoscopy
13	https://www.alberthararymd.com/contents/our-services/esophageal-dilation	https://www.healthdirect.gov.au/surgery/upper-gi-endoscopy-and-colonoscopy
14	https://www.mayoclinicproceedings.org/article/S0025-6196(12)60109-8/fulltext	https://gi.org/topics/upper-gi-endoscopy-egd/
15	https://www.endo-world.com/resources/e-learning-patient-education/procedures/esophageal-dilation/	https://www.dana-farber.org/health-library/articles/understanding-upper-endoscopy-and-colonoscopy/
16	https://www.bumrungrad.com/en/treatments/esophageal-dilation	https://stanfordhealthcare.org/medical-tests/e/egd/what-to-expect/before-procedure.html
17	https://www.dynamed.com/procedure/esophageal-dilation-and-stenting	https://myhealth.alberta.ca/Health/aftercareinformation/pages/conditions.aspx?hwid=uf9371
18	https://www.gastroenterologyandhepatology.net/archives/june-2019/esophageal-dilation-as-the-primary-treatment-for-eosinophilic-esophagitis/	https://stanfordhealthcare.org/medical-treatments/e/endoscopy/types/upper-gi.html
19	https://www.cincinnatichildrens.org/health/e/esophageal-dilatation	https://www.cancer.org/cancer/diagnosis-staging/tests/endoscopy/upper-endoscopy.html
20	https://www.atgastro.com/esophageal-dilation/	https://www.gpddc.com/2021/12/15/what-to-expect-during-and-after-upper-endoscopy/

There was minimal difference found between the “esophageal dilation” group and the “upper GI endoscopy” group. Based on an independent sample t-test, there were no significant differences in any of the readability indices between the two groups. A summary can be found in Table 4.

**Table 4:** Descriptive analysis of the readability of all sites (n=38) and the comparison of the two groups of searched websites. Statistical independent t-test analysis between group 1 and group 2 readability data showed no significant differences among the five readability indices. SMOG: Simple Measures of Gobbledygook

	Mean	Standard deviation	T-test	Minimum	Maximum	Group 1 mean	Group 2 mean
Automated Readability Index	6.8	1.6	0.1	4.1	10.3	7.3	6.4
Coleman-Liau Index	15.5	2	0.2	11.1	21.3	16	15.1
SMOG Index	7	1.2	0.1	4.4	10.2	7.3	6.6
Gunning Fog Score	8.5	1.9	0.1	5.2	13.3	9	7.9
Flesch-Kincaid Grade Level	8.2	1.7	0.1	4.9	11.8	8.7	7.7

## Discussion

Substantial evidence underscores the potential improvement of health outcomes by facilitating a better alignment between the patient comprehension of healthcare procedures and online information [18]. Specifically, individuals with limited health literacy are prone to elevated hospitalizations, poor medication adherence, and challenges following postoperative guidelines. Together, these factors lead to adverse health outcomes compared to their more educated counterparts [19]. Despite these trends, an extensive array of information available online concerning prevalent disease states in the United States persistently overshoots the national guidelines that recommend readability to be at a sixth-grade level [20]. This phenomenon mirrors the observations in the present study, highlighting that numerous information sources that contain esophageal dilation information are considerably too difficult for the general population.

Our analysis revealed that studies aligning closer to the sixth-grade reading level presented information in a more digestible manner, predominantly devoid of esoteric vocabulary and encompassing easily comprehensible medical terms. Conversely, studies that significantly surpassed the recommended reading level often employed intricate vocabulary without adequate explanations and were sparse on visual aids such as images and charts. This study elucidates that the employment of straightforward language paired with engaging visuals effectively conveys health information to the general public.

There are several ways in which digital materials can be improved. One promising approach has been the development and dissemination of instructional content through video platforms such as YouTube or more succinct mediums such as Instagram and TikTok, which have emerged as effective vehicles for simplifying complex concepts [12]. However, navigating the massive, unregulated online ecosystem presents a significant hurdle. The primary challenge is the identification of reliable sources. Recent survey data indicates that individuals with limited health literacy frequently gravitate to blogs, celebrity webpages, and unverified social media for health information [21]. In this context, it becomes clear that clinicians may consider assuming the role of an educator, guiding their patients to discern appropriate educational material. Notably, a substantial 52% of respondents in one study associated high levels of trust in specialist doctors for procuring health information [21]. Consequently, it is imperative that clinicians foster patient literacy by guiding patients to pre-vetted online resources.

Limitations

It is vital to note a few limitations of this study. Firstly, the exclusion of the UpToDate resource, generally inaccessible to the wider public, constitutes a notable drawback. Secondly, data from the Penn Medicine website was omitted due to its extreme readability index score of -7.64, categorizing it as an outlier within the dataset. Additionally, we did not use criteria when choosing websites to consider for analyses in an attempt to capture how the general patient population would access medical information based on website popularity on Google search engines. Finally, the study’s reliance on readability metrics as the singular measure poses a limitation, as it does not necessarily ensure accurate comprehension by the readers.

## Conclusions

Online material regarding esophageal dilation and upper GI endoscopy yielded an average readability score of 9.2, a score that notably surpasses the sixth-grade standard for health information by three US grade levels. Although the topic of upper GI endoscopy seems more readable than esophageal dilation, its readability nonetheless exceeds the readability benchmarks set for the wider American audience. This study highlights the pressing need for enhanced efforts to streamline the communication of health information, particularly to foster improved outcomes from esophageal dilation. As such, it is recommended that physicians adopt a more dynamic role in patient education. This strategy not only cultivates trust in healthcare professionals but also significantly amplifies the likelihood of favorable patient outcomes.
